# Non-communicable diseases in Mozambique: risk factors, burden, response and outcomes to date

**DOI:** 10.1186/1744-8603-8-37

**Published:** 2012-11-21

**Authors:** Carla Silva-Matos, David Beran

**Affiliations:** 1 Head of Non-Communicable Disease Department, Ministry of Health, Republic of Mozambique, Av. Eduardo Mondlane/Salvador Allende, Maputo, Mozambique; 2 Researcher and Lecturer, Division of International and Humanitarian Medicine, Faculty of Medicine, University of Geneva, Rue Gabrielle-Perret-Gentil 6, 1211, Geneva 14, Switzerland

## Abstract

Mozambique is located on the East Coast of Africa bordering South Africa, Zimbabwe, Zambia, Malawi and Tanzania and is one of the poorest countries in the world. Currently NCDs account for 28% of deaths in Mozambique. Risk factors such as tobacco and alcohol use and poor diet are present in both urban and rural settings. Diseases such as hypertension and diabetes affect large proportions of the population, but people are often unaware of their condition or poorly managed. Data from studies on diabetes highlight the financial burden for NCD management in Mozambique for both the individual and health system. The National Strategic Plan for the prevention and control of NCDs in Mozambique has as its aim to create a positive environment to minimise or eliminate the exposure to risk factors and guarantee access to care. The plan has as its overall objective to reduce exposure to risk factors and morbidity and mortality due to NCDs and has 4 areas of intervention: 1) Prevention and health education with regards to NCDs; 2) Access to quality care, treatment and follow-up; 3) Prevention of disability and premature mortality and 4) Surveillance, research, monitoring and evaluation and advocacy for NCDs. The Ministry of Health developed projects for diabetes and hypertension and used these as key lessons that could then be applied to other NCDs. Mozambique, through political commitment from the Ministry of Health and the dedication of local champions, has been able to garner international support to improve care for people with diabetes and then use this to develop its National Plan for NCDs. Despite this increase in attention resources available do not match the challenge of NCDs in Mozambique. Mozambique’s experience provides a practical example of actions that can be undertaken in a resource poor country to tackle the emerging burden of NCDs.

## Background

Mozambique is located on the East Coast of Africa bordering South Africa, Zimbabwe, Zambia, Malawi and Tanzania. Based on the 2007 census projections for 2011 the total population is estimated to be 23 million inhabitants [1]. The population composition of Mozambique is typical of a Low Income Country in sub-Saharan Africa with 45.3% of the population under the age of 15, 50.1% aged between 15–59 and 4.7% above the age of 60 [1]. Life expectancy (2011 estimate) is 52.4 years overall and 50.4 and 54.5 for men and women respectively [1].

Mozambique is one of the poorest countries in the world being ranked 184 out of 187 on the Human Development Index [2]. Gross Domestic Product (GDP) per capita was International US$ 1,000 and GDP (real growth) in 2010 was 7%. In parallel to this economic development the rate of urbanization was estimated to be 4% [3] with 31% of the total population living in urban areas in 2011 [1]. In comparison to other countries in Southern Africa Mozambique is currently one of the least urbanized countries [4,5]. However, by 2025 it is estimated that it will be the fourth most urbanized [4,5].

Mozambique is highly dependent on donor aid receiving US$ 87 per capita in net official development assistance and official aid [6]. An estimated 25 donors cover about 70% of Mozambique’s health budget through basket funding, with some of these donors providing direct financial assistance to the Ministry of Health and others to specific areas of the country or disease areas [7].

The prevalence of HIV/AIDS in adults aged between 15–49 years of age continues to increase at a national level and is approximately 16.1% [8]. The top 5 causes of mortality are Malaria, HIV/AIDS, Diarrhoeal diseases, Lower Respiratory Infections and Perinatal Conditions representing 57% of total deaths [9]. Despite the main causes of mortality being communicable diseases a study in 1994 showed that of a total of 8,114 deaths classified in the autopsy register in Maputo city 1,834 (22.6%) were due to non-communicable diseases (NCD) [10].

## Methods

A historical review of published and gray literature on Mozambique and NCDs was carried out. This included a review of the literature up to end of 2011 on PubMed using similar terms to those used in Echouffo-Tchegui and Kengne [11], such as “smoking”, “alcohol drinking”, “diet”, “nutrition”, “physical activity”, “obesity”, “chronic disease”, “cardiovascular diseases”, “hypertension”, “cancers”, “diabetes”, “chronic obstructive lung disease” and “asthma” in combination with Mozambique. Only articles published after 1990 were selected and no limit was placed on language of publication.

Reports and publications from the Ministry of Health, World Health Organization (WHO), disease specific organizations, such as the International Diabetes Federations (IDF), NGOs were also found and reviewed. Key documents, for example, included reports prepared by the International Insulin Foundation (IIF) on the health system and its management of diabetes, data from different cancer registries and a study on asthma from the Central Hospital in Maputo. The “response” section of this paper is based on the experience of the two authors in preparing the National NCD strategy and this work refers to this document extensively.

Statistics on the NCD risk factors and disease burden are reported from all sources equally. It is clear that these estimates may vary based on methodology, but the authors did not assess the methods or reliability of these estimates, but tried to collect as much information as possible in order to prepare a comprehensive review of the situation with regards to documenting the risk factors, burden of NCDs as well as the government response and outcomes to date.

## Risk factors

The four main NCDs, cardiovascular disease (CVD), cancer, diabetes and chronic obstructive pulmonary disease (COPD) share four common risk factors tobacco use, the harmful use of alcohol, poor diet and lack of physical activity [12]. The section below will detail these four risk factors in Mozambique including overweight and obesity. Many of the studies detailed below build on Mozambique’s use of the WHO STEPs Survey [13]. In parallel to these risk factors it is estimated that between the period 2000–2030 the population of elderly is expected to double [14] and that this is also a risk factor, albeit non-modifiable, of many NCDs.

### Tobacco use

Mozambique is a signatory to the WHO’s Framework Convention on Tobacco Control (18 June 2003), but has yet to ratify this [15]. The Global Youth Tobacco Survey found that 6.3% of students aged 13–15 had smoked a cigarette at some point in time (Male: 9.0%; Female: 3.6%) and that 10% currently use tobacco products (Male: 12.7%%; Female: 7.4%) [16]. In adults (25–64 years of age) this male:female difference also exists with the prevalence of current consumption of tobacco products in men equal to 39.9% and 18.0% in women [17]. Differences were found in different areas of the country in female consumption and in the types of tobacco products consumed. Another study found that patterns of tobacco consumption in the capital city, Maputo, were similar to the area of origin of these people [18].

### Alcohol use

In a representative sample of 3,265 Mozambicans aged 25–64 years it was found that the overall prevalence of current drinking was 28.9% in women and 57.7% in men [19]. 40% of those interviewed reported having at least one session of binge drinking in the previous week. In men prevalence of drinking increased with income and in women with income and education. In rural areas of Mozambique the use of alcohol, mostly traditional alcoholic drinks, was found in 25% of women and 50% of men [20].

### Diet

In looking at fruit and vegetable intake 31.3% of women consumed fruit 3–7 times per week in comparison to 32.7% of men [20]. For vegetables 33.0% of women consumed vegetables everyday of the week and 25.2% of men did this. In urban areas the consumption of fruit and vegetables was found to be less frequent than in rural areas [21]. The study found an increase in consumption of fruit linked with increased education, but not income. Eating vegetables was more prevalent in more educated urban men and wealthier rural women. Consumption of at least two servings of fruits was 17.8% and 18.7% for vegetables, whilst only 4.2% of people reported a daily consumption of at least five servings of fruits and vegetables per day.

### Physical activity

In a comparison between African countries with regards to meeting the WHO criteria for physical activity (at least 150 minutes of moderate activity per week or equivalent) 96.2% of people in Mozambique met this level [22]. The main contributors to physical activity in Mozambique were vigorous work, moderate work and transportation.

### Overweight and obesity

Prevalence of overweight individuals was 30.1% and 10.2% for urban and rural areas respectively [23]. Obesity rates were 11.5% in urban areas and of 2.6% in rural areas. The prevalence of obesity and overweight were 6.8% and 11.8% in women, and 2.3% and 9.4% in men. In women overweight and obesity increased with age in both urban and rural areas. Over all rates of overweight and obesity were approximately four times higher in urban than rural women. Increases in education levels (urban women and rural men) and annual income (urban and rural women and rural men) led to increases in overweight and obesity rates.

It is not only the individual risk factor that needs to be assessed, but also in some cases the cumulative effect of one risk factor versus another. Padrão et al. [20] found that tobacco use caused different consumption of fruits and vegetables as well as alcoholic drinks with for example an odds ratio of 7.3 for people smoking manufactured cigarettes and drinking beer versus non-tobacco consumers and also people who smoked ate less fruits and vegetables.

## Disease burden

NCDs account for 28% of deaths in Mozambique, of which 12% are due to CVD, 3% for cancers and respiratory diseases, 2% for diabetes and 7% for other NCDs [9]. A survey from 2001 in 4 cities in Mozambique found that the burden of NCDs varied between 13.1% and 24% of deaths [24]. NCDs in Mozambique are not only affecting adults, but are also starting to impact children. In a 10 year study of the causes of death of children under the age of 15 years in Manhica, communicable diseases were still the most prominent cause of mortality with 73.6%, but NCDs represent 13.4% of the total with 9.5% because of chronic conditions and 3.9% due to injuries [25].

### Cardiovascular diseases

Damasceno et al. [26] found a prevalence of 33.1% for hypertension in Mozambique, with only 18.4% aware of their condition. About half of those individuals aware of their condition were under treatment and control was found to be extremely low. Awareness and treatment were twice as high in women and within the sub-group of people treated the prevalence of control in women was 1.5 times higher. Linking the results of this study to the risk factors described above it was found that men and women with higher Body Mass Indexes and abdominal obesity had a higher prevalence of hypertension and that in women the prevalence of hypertension increased with the frequency of alcohol consumption. This difference was not found in men. Marijon et al. [27] ascertained, in a small study, that cardiovascular risk factors differed between ethnic groups in Mozambique with hypertension being most prevalent in the African population and this population group having very few other risk factors in comparison to the European and South East Asian groups.

This high number of people with either undiagnosed or unmanaged hypertension means that a high level of stroke is present in Mozambique. A 12 month study in Maputo using the WHO’s STEPStroke surveillance program found that 651 new stroke events occurred [28]. Translated into crude and adjusted annual incidence rates were 148.7 per 100,000 and 260.1 per 100,000 in the population aged above 25 years of age. In comparison this rate is 123.9 per 100,000 in London, United Kingdom [29]. These episodes of stroke led to high levels of mortality (in-hospital case fatality: 33.3%; 28-day case fatality: 49.6%) and disability with 64.4% of the 370 surviving individuals at 28 days having moderate to severe disability [28].

Cardiovascular disease does not only affect adults in Mozambique, but also children and adolescents. The prevalence of Rheumatic heart disease was found to be 2.3 cases per 1,000 in children aged 6 to 17 years of age living in the capital city [30]. A retrospective study of all patients cared for in a specialised referral unit looked at Congenital Heart Disease and found that of the 534 patients studied only 52.8% of these were diagnosed under the age of 2 years of age and 29.0% had complications at the time of diagnosis [31].

### Cancers

It is estimated that 3,690 women are diagnosed with cervical cancer and 2,356 die from the disease in Mozambique [32]. Cervical cancer ranks as the most frequent cancer among women aged between 15 and 44 years of age. Data from the Central Hospital in Maputo found that cervical cancer is the most common cancer in adult women representing 27.6% of all diagnosed cancers in female patients [33]. This represents an average of 78.2 cases per year. In Beira, Mozambique’s second largest city, in 2005 and 2006 cervical was the second type of cancer after Kaposi sarcoma, which are both linked to HIV/AIDS [34]. Almost 80% of these cancers were diagnosed at an inoperable phase.

In Mozambique a population based cancer registry in the city of Beira found that breast cancer was the third cause of cancer in adult women and affected those mainly aged 40 and above [35,36]. Data from the Central Hospital in Maputo found that 60% of consultations and inpatient stays in the Urology Department were related to cancers in men [37]. Lorenzoni [33] found that prostate cancer was most frequent in men aged between 65 to 74, but in young men aged under 54 years of age the most common cancer was Kaposi’s sarcoma. Of those cancers diagnosed only 5-10% were operable.

### Diabetes

Results from a STEPS survey found a diabetes prevalence of 2.9% in the general population, aged from 25 to 64 years old [38]. There were more than twice as many urban men than rural men with diabetes (prevalence of 5.5% versus 2.4%). However, in women the difference was even larger between urban and rural women with respective prevalence of 4.9% and 1.2% respectively. In 2003, the IIF estimated that there were 928 children with Type 1 diabetes, with a low life expectancy of 3.8 years in Maputo city and 7 months in rural areas [39].

As with hypertension different sources of data highlight a large proportion of people “missing” from health system data. A 2009 study found that only 27% of expected people with Type 2 diabetes were actually being followed in the health system [40]. Data from a population study found that just over 10% of people identified as having diabetes knew they had this condition [38].

Also highlighted in this study [38] and others [39-42] was the problem of diabetes management. 13% of people identified as having Type 2 diabetes were prescribed dietary changes with a further 10% also having non-pharmacological interventions such as exercise and weight loss. 9% of people used oral medicines and 3% used insulin. All those people on insulin were in urban areas. In addition a high use of traditional healers and medicine was found.

### Chronic obstructive pulmonary disease

WHO estimates that in Mozambique in 2008 there were 229 deaths per 100,000 due to chronic respiratory diseases [43]. Asthma prevalence in Mozambique is estimated at 13.3% [44] in children and adolescents.

From the total 702 asthmatic patients registered in Maputo city during a month, 100% went to emergency services at the Maputo Central Hospital and two General Hospital [45]. At the Paediatric Department of the Maputo Central Hospital, asthma is the second cause for hospitalization [46].

### Financial burden

Data from studies on diabetes highlight the financial burden for NCD management in Mozambique for both the individual and health system. For the individual in 2003 managing diabetes with insulin cost US$ 273.6 or 75% of per capita GDP [47]. For the government in an 18 month period between 2001 and 2003, the government of Mozambique spent 10% of government expenditure on medicines on insulin [42].

## How has one of the world’s poorest countries responded to the challenge of NCDs?

In Mozambique the Department for NCDs (DNCD) within the Ministry of Health was created in 2002 to address both chronic NCDs and Trauma and Violence. The work of the DNCD focused on the following different areas: [48]

1. Organisation of the Health System

2. Data Collection

3. Prevention

4. Diagnostic tools and infrastructure

5. Drug procurement and supply

6. Accessibility and affordability of medicines and care

7. Healthcare workers

8. Adherence issues

9. Patient education and empowerment

10. Community involvement and diabetes associations

11. Positive policy environment

Three studies were initiated in 2003 to gain information on different aspects of NCDs. The WHO STEPwise approach on Surveillance of NCD Risk Factors (STEPS) was implemented to get population based data on the most common risk factors for NCDs [49]. Another study focused on acute asthma at the Central Hospital in Maputo [45] and the Rapid Assessment Protocol for Insulin Access (RAPIA) was carried out in three provinces of Mozambique to assess problems with providing care and access to medicines for diabetes [42].

Besides these studies other registers and surveillance systems were developed. Questions related to injuries were introduced into the demographic study in 2003 to find incidence of injury and prevalence of disability due to injury in Mozambique [50]. An epidemiological surveillance system for injuries was also established in Maputo Central Hospital to measure the number and causes of trauma. This was then extended to other health facilities in Maputo and throughout the country. A population based cancer registry in Beira in collaboration with the International Agency for Research on Cancer was also established.

Data provided a clear picture of the burden of disease (STEPS, registers, surveillance systems) as well the existing barriers to care (RAPIA). Based on this it was decided to use diabetes and hypertension as a model for the development of an NCD Plan.

The results from the RAPIA [42] were prioritised and the main recommendations that it was decided to address were, improve and increase the role of Diabetes Association, implement Chronic Disease Law which stated that people with diabetes and other chronic conditions were entitled to 80% subsidy in their medicines, improve data collection, increase training in diabetes, improve communication between central medical store and periphery to address shortages and surpluses of insulin in some areas of the country, increase awareness of diabetes in general public, improve access to diagnostic tools, develop care guidelines adapted to the Mozambican situation and increase number of diabetes consultations.

As well as working with the Ministry of Health and clinicians to address the problems identified during the RAPIA and improve diabetes care, the DNCD actively involved the diabetes association (AMODIA) in improving care and education for people with diabetes and developing a model for a chronic consultation for both diabetes and hypertension. This collaboration also led to organising activities for World Diabetes Day from 2004 onwards. World Heart Day has also been celebrated since 2005. The aim of these events was to raise the profile and increase information on diabetes and cardiovascular disease to the general population. The organisation of these events led to increased activity in the existing diabetes association in the Capital City and the creation of new branches of this association in two other cities.

In organising these events different departments from the Ministry of Health were involved, as well as other members of civil society, the media and the community at large. This enabled the creation of an ad hoc coalition on diabetes and hypertension.

Training of health professionals was also cornerstone of developing these activities and this was done using IDF guidelines developed specifically for sub-Saharan Africa that were translated in to Portuguese and adapted to the Mozambican setting. Some training was carried out in Tanzania in collaboration with the IDF Africa Region and the Tanzanian Diabetes Association and also in Mozambique using local faculty and support from Diabetes UK and the World Diabetes Foundation (WDF) [51]. Health care workers were trained in diabetes and hypertension (2007-present) with a view of those trained to then be trainers. At present a team of healthcare workers (doctors, medical technicians and nurses) have been trained in each province in Mozambique with the responsibility of training their colleagues and developing diabetes and hypertension care in their province. The result of this was the implementation of a chronic consultation to all Central Hospitals, 2 Provincial Hospitals and 12 health centres in Maputo city. These facilities were provided with the necessary tools for the management of both hypertension and diabetes, such as blood glucose monitors, sphygmomanometer, risk tables and education materials. To date a team of healthcare workers has received training in diabetes and hypertension in each Province of the country.

Initial education materials for people with diabetes and hypertension were pamphlets developed locally describing these conditions, giving dietary and lifestyle advice as well as detailing proper measures for foot care. More detailed education materials for people with diabetes have also been developed as well as training expert patients in how to use these. These visual materials are divided into different sections which form part of different modules for education on, what is diabetes, Type 1 diabetes, Type 2 diabetes, Low blood sugar (Hypoglycaemia), High blood sugar (Hyperglycaemia), Diet, Treatment and Monitoring and control.

In looking at the issue of affordability and accessibility of insulin and medicines following the RAPIA the Chronic Disease Law was fully implemented in 2006 and then a change in government policy with the putting in place of prescription fee of US$ 0.20 in 2007. Accessibility and issues surrounding distribution were addressed by closer collaboration between the Ministry of Health, Central Medical Stores and the Provinces.

## The National Strategic Plan for the prevention and control of NCDs

The National Strategic Plan for the prevention and control of NCDs in Mozambique has as its aim to create a positive environment to minimise or eliminate the exposure to risk factors and guarantee access to care [52]. The plan which was approved by the Minister of Health in October 2008 aims to both guide local action as well as making a case why NCDs should be dealt with in Mozambique, thereby being useful as a tool for advocacy as well as a providing a framework for action. Contents of the plan are detailed in Table 1.

**Table 1:** Outline of Mozambique’s National Plan for NCDs

**Section**	**Content**
1. Introduction	a. Background information on Mozambique
	i. Geographic and demographic data
	ii. Socioeconomic information
	iii. Health indicators
	iv. Information on health system
2. Situation analysis	a. Specific disease
	i. Worldwide burden
	ii. Sub-Saharan African burden
	iii. Mozambique burden
	iv. Specific interventions for disease
3. Financial and social impact of NCDs	a. Worldwide impact
	b. Sub-Saharan impact
	c. Mozambican impact
4. Prevention strategies for NCDs	a. For specific disease
	i. Primary
	ii. Secondary
	iii. Tertiary
5. Existing measures for NCDs in Mozambique	a. Activities developed per level of prevention
	b. SWOT analysis
6. National Strategic Plan for the prevention and control of NCDs	a. Vision
	b. Mission
	c. Guiding principles
	d. General objective
	e.Strategic objectives
	f. Main strategies
	g. Areas of intervention
7. Objectives and selected strategies	
8. Implementation	a. Target group
	b. Implementation plan
9. Monitoring and evaluation	
10. Activity matrix	a. Logframes of different activities
11. Budget	

Prioritisation of which conditions to tackle through the national plan was necessary due to Mozambique’s limited resources. Through both discussions with different stakeholders and taking into account the burden of disease on the whole population, burden on the health system and also political considerations the following conditions were chosen:

Cardiovascular Disease

Hypertension

Diabetes

Cancers

Breast

Cervical

Prostate

Asthma

Table 2 summarises the data available for each condition and the reasoning behind its choice as a priority.

**Table 2:** Priority NCDs in Mozambique

**Condition**	**Data available**	**Rational for choice**
Cardiovascular disease [28]	1.7 Strokes per day in Maputo Central Hospital	High burden of disease with high mortality and burden on health system
Hypertension [26]	National prevalence of 33.1%	High burden of disease for whole population
Diabetes [38,39,42]	· National prevalence of 2.9%	High burden of disease for whole population, high cost of disease for individual and health system, high mortality for Type 1 diabetes
	· Life expectancy for Type 1 diabetes 3.8 years in Maputo City and 7 months in rural areas	
	· Between 2001–2003 expenditure on insulin represented 10% of government expenditure on medicines was spent on insulin	
Cancers		
1. Breast	1. Third cause of cancer in women based on registered cases in Beira cancer register	High level of morbidity and mortality, burden on health system
2. Cervical	2. First cause of cancer in women in cancer register in Beira, represents 27.6% of all cancers diagnosed at Maputo Central Hospital	
3. Prostate [34,36,37]	3. Data from Maputo Central Hospital shows that prostate cancer represents 60% of consultations and inpatient care in the Urology. In 2005 represented 207 cases (20%) of cancers in men	
Asthma [44-46]	· Prevalence of asthma was estimated to be 13.3% in children aged 6–7 years and adolescents aged 13–14 years.	High level of morbidity, poor management and burden on health system
	· 100% of registered asthma patients in Maputo city used emergency services during one month	
	· In paediatric department asthma is second cause of inpatient treatment	

The plan has as its overall objective to reduce exposure to risk factors and morbidity and mortality due to NCDs and has 4 areas of intervention:

1. Prevention and health education with regards to NCDs.

2. Access to quality care, treatment and follow-up.

3. Prevention of disability and premature mortality.

4. Surveillance, research, monitoring and evaluation and advocacy for NCDs.

In order to effectively implement this NCD plan throughout the country focal points have been nominated in all provinces. Their role will be to implement the plan in their provinces adapting the guiding principles of the plan to their local setting.

For each of these areas of intervention certain strategies and key interventions have been chosen. These interventions are both population and individual interventions and build on existing strengths within the Mozambican Health System.

### Prevention and health education with regards to NCDs

This area of intervention focuses primarily on increasing knowledge and awareness of NCDs and healthy lifestyles. The plan will aim to do this by developing a communication strategy for NCDs that will help increase this knowledge and awareness to many stakeholders in Mozambique, from donors, the Ministry of Health, other Ministries and Mozambicans. Primary, secondary and tertiary prevention will all be included in this area of intervention. Education using Information, Education and Communication (IEC) messages and materials will be combined with an integrated package of materials for counselling for patients and their family to improve their capacity and competency to manage NCDs. Civil society will play an active role in all of these activities. This will build on and be integrated with other existing programmes, e.g. Sexually Transmitted Diseases, vaccination programmes, etc.

As many factors impacting NCDs fall outside of the realm and responsibilities of the Ministry of Health, the Ministry will lead advocacy for the establishment of a positive legislative and political environment for the prevention of NCDs. In addition legislation will be developed to ensure free access to medicines and care for people with NCDs.

The key element of this area of intervention is the involvement of different partners and stakeholders to address the challenge of NCDs.

### Access to quality care, treatment and follow-up

Training will play an important role in ensuring access to quality care, treatment and follow-up. This will focus on pre and post graduate training by developing plans and manuals for training. This training will take an overall perspective on NCDs focusing not only on clinical care, but also primary prevention, counselling, patient education and rehabilitation.

This will be done by building collaborations with training institutions and promoting on the job training for all aspects of NCDs.

### Prevention of disability and premature mortality

Organising the health system around the management of NCDs is a crucial step in guaranteeing the prevention of disability and premature mortality. The health system will be reoriented to primary and secondary levels. This will include prevention, screening and rehabilitation services with the ensuring appropriate referral and counter-referrals. Norms and protocols will be developed for this and detail the roles and responsibilities of each level of the facilities. These will include lists of equipment and medicines adapted to all levels of attention including the private sector. In order to achieve this one main project will be to improve the supply of medicines and equipment for NCDs as well as provide training for laboratory and pharmacy personnel.

### Surveillance, research, monitoring and evaluation and advocacy for NCDs

Data is indispensable to assess the implementation of the national plan as well as help with advocacy activities. This data will be collected through implementation of NCD registers and surveillance systems. These will be integrated with communicable disease surveillance systems. Another source of data will be studies. These studies will not only be for primary data collection, such as a study on the economic impact of NCDs on individuals and their families, but also to assess new interventions, such as a planned feasibility study for the introduction of HPV vaccination.

Monitoring and evaluation will be strengthened for NCDs. Included in this will be re-implementing the WHO STEPs assessment and RAPIA to assess the impact of preventive strategies and changes in risk factors and the impact of the projects and programmes carried out to date.

In addition to this overall strategy the Ministry of Health is also proposing specific measures on some of the NCD risk factors, such as alcohol [53].

## Lessons from diabetes and hypertension

By developing projects for diabetes and hypertension Mozambique was able to develop a series of key lessons that could then be applied to other NCDs. The success of World Diabetes Day and World Heart Day activities in raising awareness and involving different areas of the health system and departments of the Ministry of Health has been replicated with the creation of “Health Days” addressing the shared risk factors of the most common NCDs as well as including education of self examination for breast cancer. Different information and education materials for this have been developed. The departments of School Health, Women’s Health, Nutrition and Medical Services have been involved in these activities and will play an active role in implementing the plan.

The diabetes and hypertension consultation provides not only laboratory testing, a clinical consultation, but also education, psychological counselling and community support. This model will be developed for all NCDs set out as priorities in the plan, involving patient associations and civil society where possible. For example women’s groups have already been approached to involve them in breast and cervix cancer activities.

Training of healthcare workers was key to the success of this project. It is planned to organise training for a range of healthcare workers and have them act as trainers in their respective provinces. Also specific roles for different levels of health professionals will be defined for each type of condition.

In parallel to trained healthcare workers the necessary tools for screening and treatment of the priority NCDs will be necessary. Toolkits for each condition have been developed and a reevaluation using the RAPIA (2009) [41] enabled the assessment if the measures that were implemented to improve access to medicines and diagnostic tools for diabetes have been successful or not and learn from this experience.

In addition integration was started between some aspects of care with the HIV/AIDS programme and diabetes and hypertension with regards to testing, counselling services and homecare. This was done by integrating diabetes and hypertension into the training manuals for healthcare workers working in the area of HIV/AIDS.

### Outcomes to date

Some of the outcomes from the plan have been the nomination by the Minister of Health in all provinces of NCD focal points in order to adapt the guiding principles of the plan to their local setting. Also within the Ministry of Health the organisation of “health fairs” enabled the creation of a coalition on NCDs including departments from the Ministry of Health, members of civil society, the media and the general population. This also strengthened and developed the diabetes association by increasing its visibility in the community.

With regards to the health system, consultations for diabetes and hypertension were established in 2006 with all 3 Central Hospitals, 7 Provincial Hospitals, 12 Health Centres in Maputo and two Provincial Health Centres having functioning consultations by the end of 2009. Other improvements in supply and availability of insulin, availability of diagnostic tools and trained healthcare workers have also been seen [41]. For diabetes specifically this has led to an increase in estimated life expectancy [41]. Breast and cervical cancer screening programmes have been integrated with family planning services in 2010.

In addition to this NCDs were included in two key government documents. NCDs are mentioned in Mozambique’s “Plano de Acção para a Recução da Pobreza Absoluta II” (PARPA, Poverty Reduction Plan) in that their burden is increasing and that Mozambique is facing a double burden of diseases [54]. The Plano Económico e Social (Economic and Social Plan) of Ministry of Health also includes NCDs and the activities that have been carried out with regards to all NCDs and diabetes [8]. This document is the annual plan for Ministry of Health based on the PARPA and sets the activities for the different areas of the Ministry.

## Conclusion

Mozambique, through political commitment from the Ministry of Health and the dedication of local champions, has been able to garner international support to improve care for people with diabetes dramatically and then use this to develop its National Plan for NCDs.

Despite this increase in attention resources available do not match the challenge of NCDs in Mozambique. The budget for 2010 for the NCD Department at MISAU was US$ 97,000 up from about US$ 73,000 for 2009. The whole budget is provided by the government budget as no donors contribute to NCDs. This amount represents about 2.2% of the total budget for the National Directorate of Public Health. Financial assistance from the World Diabetes Foundation, Diabetes UK and WHO has been a key factor in advancing planning solutions to address various aspects of this growing challenge. External support totalled approximately US$ 160,000 per year from 2003–2009 to implement this National Plan highlighting that addressing NCDs in resource poor settings is possible with a low level of investment, but more funding is necessary. In comparison the Global Fund has approved a total of US$ 393,141,245 for HIV/AIDS, TB and Malaria projects [55] as well PEPFAR providing US$ 269,100,000 in 2010 [56]. In 2007 PEPFAR activities in Mozambique focused solely on aspects around HIV/AIDS treatment and prevention including healthcare worker training and institutional capacity [57]. This highlights the discrepancy in funding at a country level that also exists on an international level [58].

Having the 4 areas of intervention assisted in linking with other programmes aimed at these different levels of prevention, e.g. school health, women’s health, nutrition, etc. The inclusion of data and advocacy activities in the plan will assist in both monitoring the implementation of the plan as well gathering data and to aid in developing the plan further as well as informing key stakeholders and partners about NCDs. Having NCD focal points in each Province means that this National Plan will be implemented in the whole country and adapted to local realities.

To assist with garnering support data collection through studies and routine data collection tools was essential. Using diabetes and hypertension as a model for NCDs allowed Mozambique to implement projects whilst developing its National Plan and learn from these examples. With the key lessons being training, increasing public awareness, involving civil society and developing education materials for people with diabetes.

Integration between NCDs and with other diseases is essential. Integration with services for HIV/AIDS will be done with counselling services and also palliative care. For NCDs Mozambique has already integrated hypertension and diabetes. Another example of this combination of conditions is improving rehabilitation services which will not only benefit victims of road traffic accidents, but also people who have suffered a stroke and amputations due to diabetes

The implementation of the Mozambican National NCD Plan provides a practical example of actions that can be undertaken in a resource poor country to tackle the emerging burden of NCDs. Data collection on the one hand provided a clear picture of the burden of disease (STEPS, registers, surveillance systems) as well the existing barriers to care (RAPIA). Using diabetes and hypertension as a model for NCDs allowed Mozambique to implement projects whilst developing its National Plan and learn key lessons about the importance of training, increasing public awareness, involving civil society and developing education materials.

## Competing interests

The authors declare that they have no competing interests.

## Authors’ contributions

Both CSM and DB worked on all aspects of this paper from drafting to final submission. Both authors read and approved the final manuscript.
